# Worse prognosis of local and locally advanced head and neck Merkel cell carcinoma: Is it time to change the treatment paradigm?

**DOI:** 10.3389/fimmu.2025.1691000

**Published:** 2025-11-21

**Authors:** Ronen Brenner, Hanna T. Frumin Edri, Ina Sarel, Anna Levko, Sofiia Turaieva, Tanzilya Tairov, Ilia Berezhnov, Shlomit Fenig, Eyal Fenig, Tomer Ziv-Baran, Alexander Yakobson, Walid Shalata

**Affiliations:** 1 Edith Wolfson Medical Center, Oncology Institute, Holon, Israel; 2 Faculty of Medical and Health Sciences, Tel-Aviv University, Ramat-Aviv, Tel-Aviv, Israel; 3 NurExone Biologic Ltd, Ramat Gan, Israel; 4 Institute of Oncology, Kaplan Medical Center, Faculty of Medicine, Hebrew University, Jerusalem, Israel; 5 Institute of Oncology, Davidoff Center, Rabin Medical Center, Beilinson Hospital, Petah Tikva, Israel; 6 Department of Epidemiology and Preventive Medicine, School of Public Health, Faculty of Medicine, Tel Aviv University, Tel Aviv, Israel; 7 The Legacy Heritage Cancer Center and Dr. Larry Norton Institute, Soroka Medical Center, Beer Sheva, Israel; 8 Medical School for International Health, Faculty of Health Sciences, Ben Gurion University of the Negev, Beer Sheva, Israel

**Keywords:** Merkel cell carcinoma, head and neck, prognosis, neoadjuvant immunotherapy, survival analyses, treatment paradigm, Merkel cell polyomavirus

## Abstract

**Background:**

Merkel cell carcinoma (MCC) is a rare, aggressive neuroendocrine skin cancer with high metastatic potential. The impact of primary tumor location on survival outcomes for local and locally advanced disease remains incompletely understood, particularly regarding the influence of chronic sun exposure.

**Objective:**

To investigate the association between primary tumor location and overall survival in patients with local and locally advanced MCC, and explore the newer implications for treatment strategy.

**Methods:**

We conducted a multicenter retrospective analysis of Israeli patients with non-metastatic MCC with long-term follow-up. Overall survival was assessed by primary tumor location (head and neck versus other sites) using Kaplan-Meier analysis and Cox proportional hazards models adjusted for age, gender, and TNM stage.

**Results:**

In total, 191 patients with local and locally advanced MCC were included, of whom 64 had head and neck MCC and 127 had MCC at other anatomical sites. Primary tumors located in the head and neck region were associated with significantly worse 5-year overall survival (51.6%) compared to other anatomical sites combined (65.2%, p = 0.025). In multivariate analysis, head and neck locations were associated with a significantly increased mortality risk (HR = 1.769, 95% CI: 1.104–2.835, p = 0.018) after controlling for age, gender, and TNM stage.

**Conclusion:**

Local and locally advanced head and neck MCC carries a significantly worse prognosis compared to MCC at other anatomical sites. Recent evidence of favorable responses to neoadjuvant immunotherapy in MCC, coupled with our findings, suggests that patients with head and neck disease may be appropriate candidates for this novel treatment approach. A paradigm shift toward neoadjuvant immunotherapy, especially for head and neck MCC, warrants serious consideration.

## 1 Introduction

Merkel cell carcinoma (MCC) is a rare, aggressive neuroendocrine skin cancer with a high propensity for local recurrence, regional spread, and distant metastasis. Despite its rarity, with an annual incidence of approximately 0.7 per 100,000 person-years in the United States, the five-year survival rate for Merkel cell carcinoma (MCC) is approximately 79% for localized disease, 66% for regional spread, and drops to 31% for distant metastases (1–3), the incidence of MCC has increased dramatically in recent decades, with a four-fold rise between 1986 and 2011 (2, 3). This upsurge is likely attributable to aging populations, improved diagnostic techniques, greater awareness among clinicians, increased UV exposure, Merkel cell polyomavirus exposure, and higher iatrogenic immunosuppression (4–9). MCC predominantly affects elderly individuals with light skin phototypes, with a median age at diagnosis of approximately 75–80 years and a male predominance (10–12). The etiopathogenesis of MCC involves multiple factors, with two primary pathways identified (13). The first pathway involves Merkel cell polyomavirus (MCPyV) infection, which is detected in approximately 80% of tumors in Western countries (14–16). The second pathway relates to cumulative ultraviolet radiation exposure leading to high mutational burden and UV-signature mutations (17, 18). The role of UV radiation is particularly significant, as evidenced by the characteristic distribution of primary tumor sites, with approximately 40–50% occurring in the chronically sun-exposed head and neck region, while others develop on intermittently sun-exposed areas of the extremities, or more rarely in sun-protected sites (7, 10, 19). The treatment approach for localized or locoregionally advanced MCC has evolved over time and now involves wide local excision with consideration of sentinel lymph node biopsy, resection of nodal disease, followed by radiation therapy and chemotherapy in selected cases (20–23). The incorporation of immunotherapy as a treatment modality for MCC represents a significant advancement in recent years (24, 25). Immunotherapy became the standard treatment for metastatic disease, but its role in the treatment of non-metastatic disease is still evolving. The current treatment paradigm does not account for the potentially significant impact of primary tumor location on prognosis, which might warrant a more tailored therapeutic approach. Emerging evidence suggests that the primary tumor site has significant prognostic implications, with several studies reporting inferior outcomes for all stages MCC with head and neck primaries compared to disease with other sites of origin (26, 27). Recently, clinical trials have demonstrated promising results of response rates and survival with neoadjuvant immunotherapy in MCC (28, 29), raising the question of whether patients with local or locally advanced head and neck MCC face a worse prognosis and should be considered primary candidates for neoadjuvant approaches. Our study aimed to evaluate the impact of primary tumor location on survival outcomes in patients with a specific group of local and locally advanced MCC, with a particular focus on head and neck disease, and to explore the implications for evolving treatment paradigms in this high-risk subpopulation.

## 2 Materials and methods

### 2.1 Study participants

Patients were aged 18 years or older at diagnosis. both sexes (male and female) were included in the study. None of the patients had undergone previous systemic therapy for local, advanced or metastatic diseases. Each patient underwent evaluation and assessment by a multidisciplinary team of specialists, including general medical and radiation oncologists, dermatologists, plastic surgeons, nuclear physicians, and pathologists. The treatment plan was personalized based on the patient’s unique condition, pathology, and imaging findings, with a primary physician overseeing and managing the entire treatment process.

### 2.2 Participants excluded

Patients were excluded from the study if their medical records were incomplete or lacked essential information regarding their medication history. Furthermore, individuals who had previously received any form of systemic therapy for localized, advanced, or metastatic disease were considered ineligible. This included those who had undergone chemotherapy, tyrosine kinase inhibitors, immunotherapy, or any other type of systemic cancer treatment at any point. These exclusion criteria were applied to minimize potential confounding factors and ensure that the study results accurately reflected the impact of first-line therapy.

### 2.3 Study design

This study analyzed a cohort of Israeli patients diagnosed with MCC between September 1985 and February 2024, collected from three major medical centers: Rabin Medical Center, Soroka Medical Center, and Wolfson Medical Center. Of the 236 patients identified, 191 with local or locally advanced disease and complete clinical data were included in the analysis (23). All patients had histologically confirmed MCC with clinical data including primary tumor location, staging information, and long-term follow-up. Patients with metastatic disease at presentation were excluded. The study was conducted in accordance with the Declaration of Helsinki and approved by the Institutional Review Board of each participating institution. Patient consent was waived due to the retrospective nature of the study and the use of de-identified data.

### 2.4 Data collection

Demographics, clinical characteristics, treatment modalities, and survival outcomes were retrospectively collected from medical records. Primary tumor locations were categorized as head and neck (chronically sun-exposed) or other locations (including trunk, upper extremities, and lower extremities). Staging was performed according to the American Joint Committee on Cancer (AJCC) 8th edition staging system for MCC (10). Patients with insufficient records or no follow-up data were excluded from the analysis. Overall survival (OS) was defined as the time from diagnosis to death from any cause or last follow-up. Disease-free survival (DFS) was defined as the time from diagnosis to first recurrence, death from any cause, or last follow-up. Death records were obtained from the Israel Ministry of Interior. The follow-up was censored on 1 February 2024 (23).

### 2.5 Statistical analysis

Continuous variables were expressed as median with interquartile range (IQR) and compared using the Mann-Whitney U test. Categorical variables were expressed as frequencies and percentages and compared using the chi-square test or Fisher’s exact test as appropriate. Survival analyses were performed using Kaplan-Meier methods, and differences between groups were compared using the log-rank test. Multivariate analyses using Cox proportional hazards models were conducted to identify independent prognostic factors while controlling for age at diagnosis, gender, location, and TNM stage. Hazard ratios (HR) with 95% confidence intervals (CI) were calculated. The proportional hazards assumption was verified using Schoenfeld residuals. All statistical analyses were performed using SPSS version 28.0 (IBM Corp., Armonk, NY, USA). Statistical significance was set at *p* < 0.05, and all tests were two-sided.

## 3 Results

### 3.1 Patient characteristics

The entire cohort consisted of 236 MCC patients. Of these, 191 patients with local and locally advanced MCC with complete clinical data were included in the study, comprising 64 patients with head and neck MCC and 127 patients with MCC at other anatomical sites. The baseline characteristics of the overall cohort (n = 191) were as follows: median age at diagnosis was 73.17 years (IQR: 64.08–81.7), with a male predominance (57.1%). The majority of patients, 133(69.6%), received radiation therapy, with a median dose of 47 Gy (IQR: 45–50); 163 patients (85.3%) underwent surgery, and 78 (40.8%) received chemotherapy.

### 3.2 Comparison between head & neck and other sites

Significant differences were observed between head and neck MCC and other anatomical sites (**Table 1**). Head and neck patients were significantly older at diagnosis (median 76.3 *vs*. 71.7 years, *p* = 0.039) and less likely to undergo surgery (76.6% *vs*. 89.8%, *p* = 0.015). Moreover, when stratifying by age, the poorer prognostic impact of head and neck location was evident in both younger and older patients. Among those younger than 76 years, head and neck location were significantly less frequent (43.8%) compared with other sites (62.2%, p = 0.02). Similarly, in patients aged 76 years or older, head and neck location were significantly more frequent (56.2%) than in other sites (37.8%, p = 0.02). This indicates that the significantly poorer prognosis associated with head and neck location holds true across both age groups. The T stage distribution differed significantly (*p* = 0.004), with T1 tumors more common in head and neck (65.6%) compared to other sites (40.9%).

**Table 1:** Comparison of characteristics between head & neck and other sites MCC.

Characteristic	Head & neck (n = 64)	All other sites (n = 127)	*P*-value
Age at diagnosis, median (IQR) [years]	76.27 [66.28–85.55]	71.73 [61.58–80.53]	0.039
<76 years	28 (43.8%)	79 (62.2%)	0.02
≥76 years	36 (56.2%)	48 (37.8%)	0.02
Male (%)	38 (59.4)	71 (55.9)	0.647
Received radiation (%)	43 (67.2)	90 (70.9)	0.602
Radiation dose, median (IQR) [Gy]	48 [41–52]	46 [45–50]	0.751
Had surgery (%)	49 (76.6)	114 (89.8)	0.015
Had chemotherapy (%)	26 (40.6)	52 (40.9)	0.966
SLNB (%)	42 (65.6)	71 (55.9)	0.197
T stage			0.004
T1	42 (65.6)	52 (40.9)	
T2	10 (15.6)	45 (35.4)	
T3	4 (6.3)	10 (7.9)	
N stage			0.666
N0	33 (55.0)	64 (51.6)	
N1, N2	27 (45.0)	60 (48.4)	
TNM stage			0.401
Stage 1	27 (42.2)	41 (32.3)	
Stage 2	11 (17.2)	25 (19.7)	
Stage 3	26 (40.6)	61 (48.0)	

IQR, interquartile range; SLNB, sentinel lymph node biopsy.

### 3.3 Overall survival analysis and disease-free survival analysis

**Figure 1:**
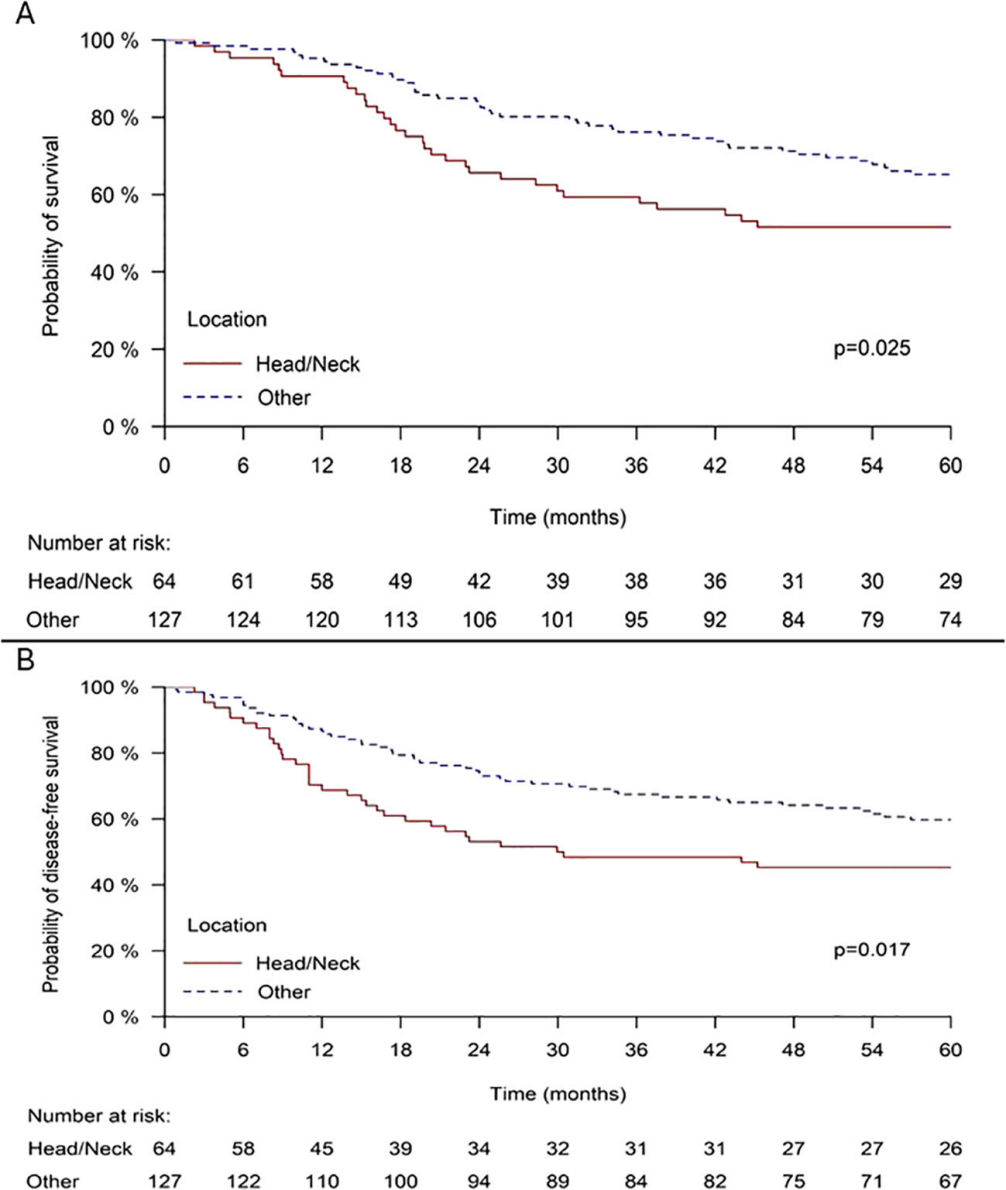
**(A)** Overall survival in head/neck MCC patients, **(B)** Disease free survival in head/neck MCC patients.

Head and neck MCC demonstrated significantly worse overall survival compared to other anatomical sites. The 5-year OS for head and neck tumors was 51.6% versus 65.2% for all other sites combined (*p* = 0.025) (**Figure 1A**). Disease-free survival followed a similar pattern, with head and neck MCC showing significantly worse outcomes. The 5-year DFS for head and neck tumors was 45.3% compared to 59.8% for other sites (*p* = 0.017) (**Figure 1B**).

In multivariate analysis, controlling for age, gender, and TNM stage, head and neck location was associated with significantly worse DFS (HR = 1.806, 95% CI: 1.160–2.811, p = 0.009), and it was associated with significantly worse survival (HR = 1.769, 95% CI: 1.104–2.835, *p* = 0.018). Male gender and increasing age at diagnosis were also independently associated with worse survival (**Table 2**).

**Table 2:** Multivariate Cox regression analysis for disease-free survival and overall survival.

Variable	Disease-free survival			Overall survival		
	HR	95% CI	*P*-value	HR	95% CI	*P*-value
Head & Neck location	1.806	1.160–2.811	0.009	1.769	1.104–2.835	0.018
Male gender	1.647	1.027–2.641	0.039	1.865	1.113–3.124	0.018
Age (per year)	1.06	1.037–1.083	<0.001	1.074	1.048–1.100	<0.001
TNM Stage			0.069			0.191
I	Ref			Ref		
II	1.867	1.003–3.473	0.049	1.485	0.763–2.888	0.244
III	1.8	1.944–3.104	0.034	1.709	0.955–3.058	0.071

HR, hazard ratio; CI, Confidence Interval.

When patients with unknown primary sites were excluded (n = 171), the survival difference persisted with head and neck 5-year OS of 51.6% versus 64.2% for other sites (*p* = 0.038).

### 3.4 Survival by anatomical site

**Figure 2:**
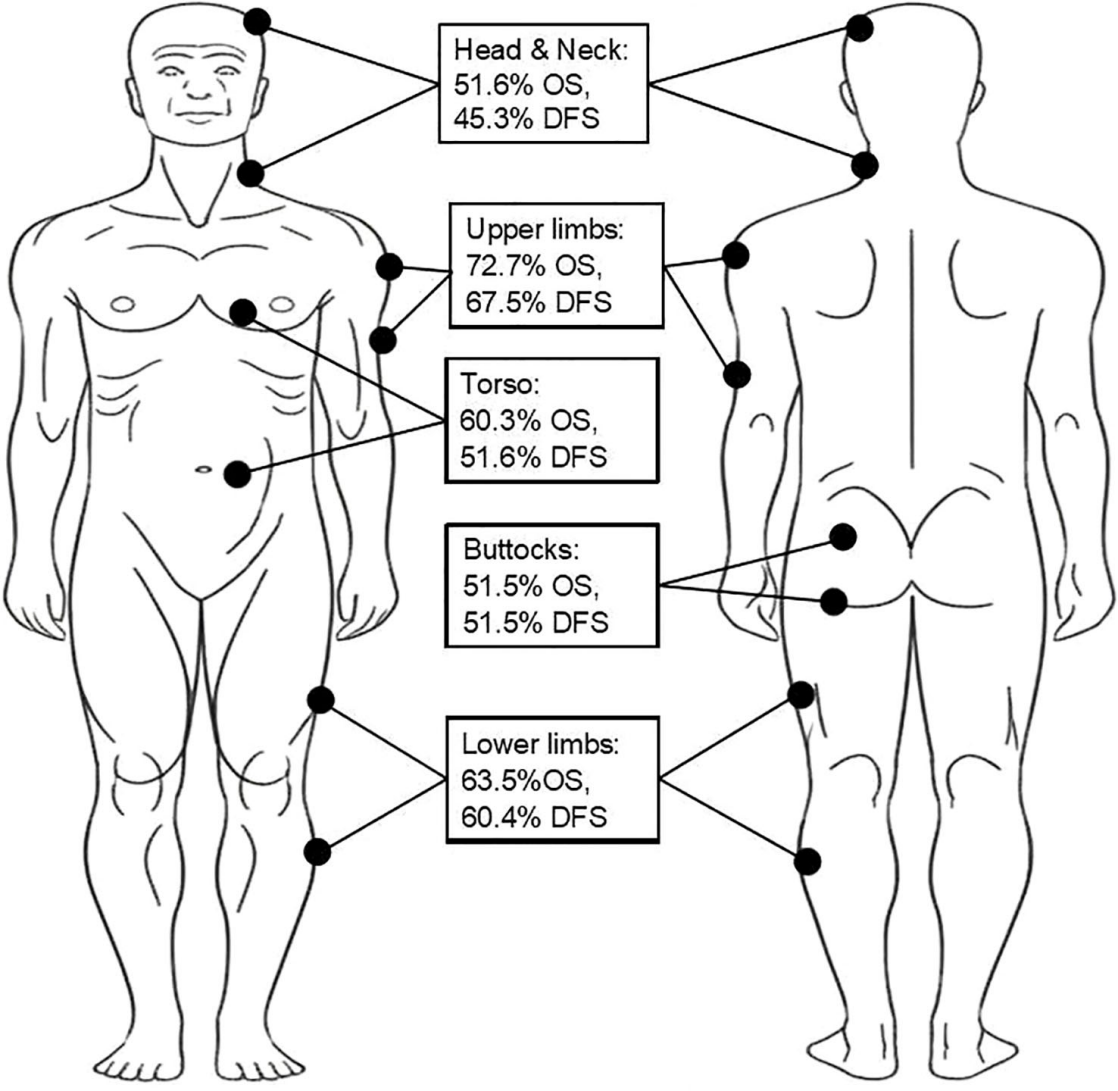
Five-year overall survival (OS) and disease-free survival (DFS) rates of patients, stratified by anatomical tumor location.

When examining survival by specific anatomical locations, head and neck demonstrated the poorest outcomes (**Figure 2**):

- Head & Neck: 51.6% 5-year OS (45.3% DFS)
- Upper limbs: 72.7% 5-year OS (67.5% DFS)
- Lower limbs: 63.5% 5-year OS (60.4% DFS)
- Torso: 60.3% 5-year OS (51.6% DFS)
- Buttocks: 51.5% 5-year OS (51.5% DFS).

### 3.5 Impact of surgery

**Figure 3:**
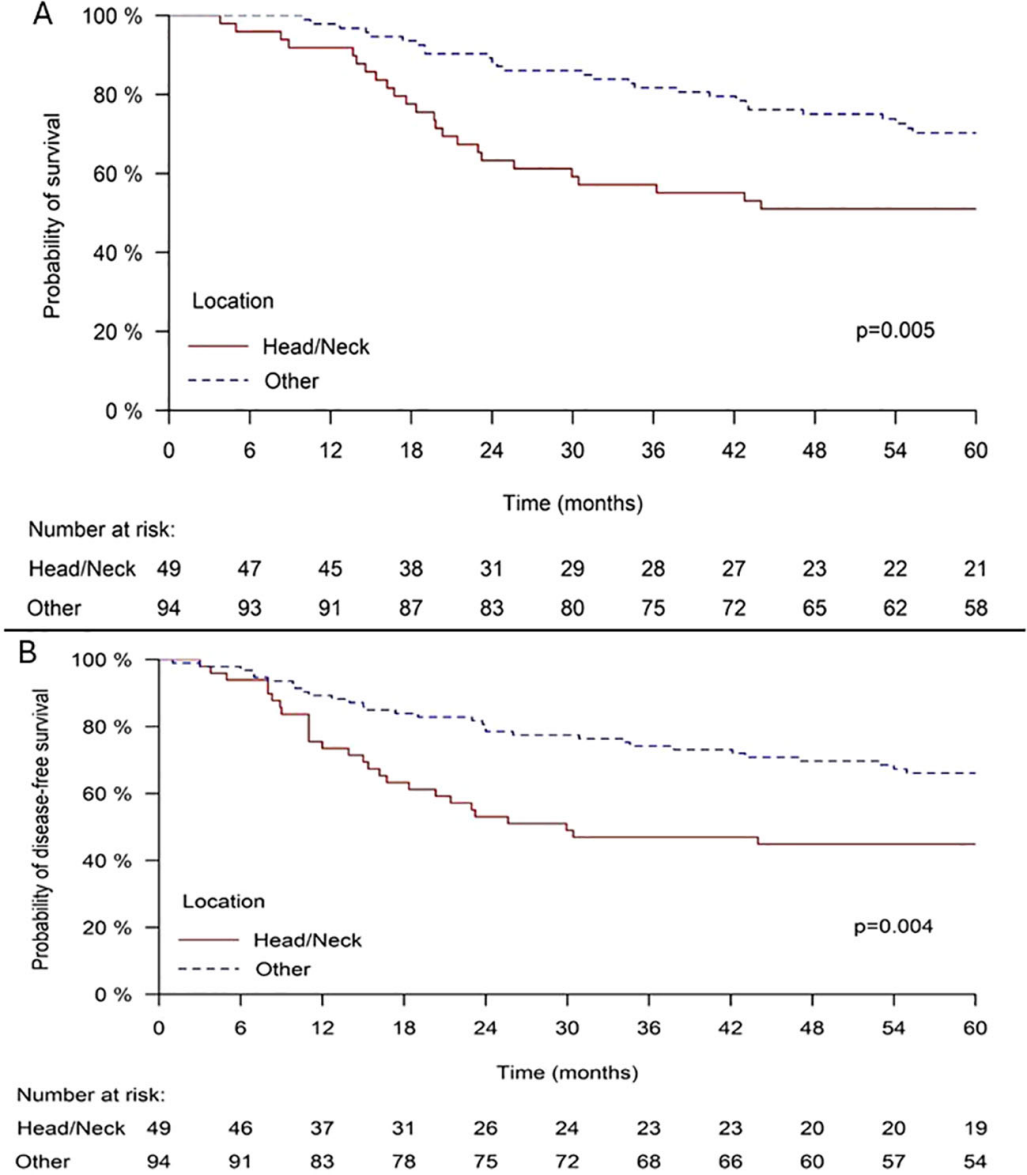
**(A)** Overall survival in MCC patients who had surgery, according to the tumor location. **(B)** Disease free survival in MCC patients who had surgery, according to the tumor location.

While surgery was associated with improved outcomes overall (64.4% *vs*. 38.7% 5-year OS without surgery, *p* = 0.004), the adverse impact of head and neck location also persisted among surgical patients. In the surgical cohort, head and neck patients continued to show worse survival (51.0% *vs*. 70.2% 5-year OS, *p* = 0.005) (**Figure 3A**) and disease-free survival (44.9% *vs*. 66.1% 5-year DFS, *p* = 0.004) (**Figure 3B**) compared to other sites. In multivariate analysis of surgical patients, head and neck location was the strongest adverse prognostic factor for both OS (HR = 2.884, 95% CI: 1.598–5.207, *p* < 0.001) and DFS (HR = 2.469, 95% CI: 1.435–4.248, *p* = 0.001), (**Table 3**).

**Table 3:** Multivariate Cox regression analysis for overall survival and disease-free survival in patients who had surgery.

Variable	Overall survival			Disease free survival		
	HR	95% CI	*P* value	HR	95% CI	*P* value
Head & Neck location	2.884	1.598-5.207	<0.001	2.469	1.435-4.248	0.001
Male Gender	2.406	1.313-4.408	0.004	2.272	1.287-4.012	0.005
Age at diagnosis	1.072	1.043-1.102	<0.001	1.059	1.034-1.085	<0.001
TNM stage	1.840	1.006-3.367	0.048	1.778	1.010-3.130	0.046

HR, hazard ratio; CI, Confidence Interval.

## 4 Discussion

Our study provides evidence that head and neck location is an independent adverse prognostic factor in local and locally advanced MCC. Patients with head and neck MCC demonstrated significantly worse 5-year overall survival (51.6%) compared to those with MCC at other body sites (65.2%), representing an absolute survival difference of 13.6%. This finding persisted in multivariate analysis, controlling for established prognostic factors such as age, gender, and TNM stage, with head and neck tumors showing a significant increase in mortality risk (HR = 1.769). DFS is an important adjunctive parameter (for measurement of the specific disease impact) and showed the same pattern with a significant 14.5% difference. These differences were even larger for patients who underwent surgery, with absolute differences of 19.2% and 21.2% for OS and DFS, respectively. Our study examines the impact of primary tumor location on the prognosis of patients with non-metastatic MCC. It supports and reinforces previous studies of cohorts, mostly including both non-metastatic and metastatic MCC, which have shown worse outcomes for head and neck MCC, which also may be related to the age of the patients, to perform surgery with correct margins and the location itself. An analysis by Zaidi et al. of over 7800 MCC cases from the National Cancer Database found that all stages head and neck tumors were associated with significantly increased mortality compared to those of the extremities, with adjusted hazard ratios of 1.306 (95% CI: 1.190–1.433) for head/neck versus upper extremity, and 1.319 (95% CI: 1.180–1.473) for head/neck versus lower extremity (26). Elias et al. reported significantly lower survival for all stages head and neck MCC versus other body primaries with five-year overall survival of 38.7% compared to 47.3% (*p* < 0.001) and even poorer outcomes specifically for scalp and neck primaries, with five-year overall survival of just 29.7% compared to 41.3% for other head and neck sites (30). Additional studies have corroborated these findings, with Morand et al. demonstrating poorer outcomes for head and neck MCC in their analysis (26, 27, 31). Several proposed mechanisms could explain these consistent prognostic differences. The marked difference in MCPyV status according to anatomic location is particularly significant. Studies have demonstrated that MCPyV status varies significantly by tumor location, with a substantially higher proportion of virus-negative tumors in the head and neck region. Placke et al. reported that only 46.2% of head and neck MCC tumors were MCPyV-positive, in contrast to tumors on the trunk and extremities, where 92.3% were virus-positive (*p* = 0.003) (32). This distribution pattern has been corroborated by multiple studies, including Harms et al., who found that virus-positive tumors are significantly more common on extremities, while sun-exposed areas, particularly the head and neck, have higher rates of virus-negative MCC (33). The clinical significance of this relationship becomes apparent when examining outcomes, as MCPyV-negative tumors consistently demonstrate more aggressive behavior. This observation is linked to the significantly higher tumor mutational burden of virus-negative MCC, nearly 100-fold greater than that of virus-positive tumors, reflecting extensive UV-induced DNA damage (17). Goh et al. documented distinct mutational signatures in virus-negative tumors that correspond to UV damage and genomic instability (18). Virus-negative MCC also typically shows TP53 and RB1 mutations, whereas virus-positive tumors are driven by viral oncoproteins, potentially resulting in different clinical behaviors (34). Harms et al. demonstrated that these different oncogenic mechanisms lead to distinct tumor biology despite similar clinical presentations (35). Another potential mechanism related to this difference is that chronically sun-exposed skin exhibits altered immune surveillance due to years of UV-induced immunosuppression, which may facilitate immune evasion (36). This chronic immunosuppression may create a permissive microenvironment for more aggressive tumor growth in the head and neck region. The complex lymphatic drainage of the head and neck region may also contribute to earlier dissemination of disease. Studies demonstrate that anatomical location significantly influences patterns of nodal metastasis and subsequent survival in cutaneous malignancies of the head and neck (37, 38). Given the significantly poorer prognosis of local and locally advanced head and neck MCC established by our study and reinforced by earlier data, this higher-risk patient population is in special need of exploring newer alternative treatment approaches. The recent emergence of immunotherapy, particularly in the neoadjuvant setting, represents a promising strategy that may address the unique challenges posed by local and locally advanced head and neck MCC. Recent clinical trials have demonstrated impressive results with neoadjuvant immunotherapy in MCC. The phase I/II CheckMate 358 trial, the first study of neoadjuvant anti-PD-1 therapy for resectable MCC, showed that among 36 patients with stage IIA-IV resectable MCC who underwent surgery after receiving nivolumab, 47.2% achieved a pathologic complete response (pCR) (28). Importantly, radiographic tumor reduction of ≥30% was observed in 54.5% of evaluable patients who underwent surgery, and no patient with a pCR experienced tumor relapse during follow-up (median 20.3 months). Further support for the neoadjuvant approach comes from a recent National Cancer Database analysis examining neoadjuvant immunotherapy in MCC patients with clinically detected regional lymph node metastasis (29). Among 1809 patients with clinically node-positive disease, 356 (19.7%) received neoadjuvant immunotherapy followed by surgical excision. The rate of complete pathologic response for the primary tumor was 45.2%, remarkably similar to the findings in the CheckMate 358 trial. When propensity-matched analysis was performed, a significant overall survival benefit was observed with neoadjuvant immunotherapy (median not reached *vs*. 35.0 months; *p* = 0.025), with a 5-year OS of 57% in the neoadjuvant immunotherapy group versus 44% in the standard approach group (*p* = 0.021), representing a substantial 13% absolute survival benefit. Notably, the utilization of neoadjuvant immunotherapy for MCC has increased dramatically in recent years, from just 0.5% in 2012 to 56.9% in 2019, reflecting growing recognition of its potential benefits (29). This trend parallels the evolution of immunotherapy in other aggressive cutaneous malignancies, such as melanoma, where neoadjuvant approaches have demonstrated improved event-free survival in randomized trials (39, 40). Current data on advanced or metastatic MCC demonstrate high objective response rates of 40–60% with anti-PD-1/PD-L1 agents as first-line therapy (24, 41). This high response rate in the metastatic setting suggests that MCC is particularly susceptible to immune checkpoint blockade, further supporting the neoadjuvant approach. Nghiem et al. demonstrated durable responses to pembrolizumab in advanced MCC, with 3-year survival rates of 89.5% among responders, highlighting the potential for long-term disease control with these agents (42). The biological rationale for neoadjuvant immunotherapy is particularly compelling for head and neck MCC. While these tumors are more frequently virus-negative and generally associated with worse outcomes, their high mutational burden may create a rich neoantigen landscape that can be effectively targeted by immunotherapy when administered in the neoadjuvant setting (43). By initiating immune checkpoint inhibition while the primary tumor and all its potential antigens remain in place, neoadjuvant immunotherapy may generate a more robust and diverse anti-tumor immune response, potentially overcoming the inherently more aggressive biology of these tumors (44). Interestingly, the CheckMate 358 trial found that responses to neoadjuvant nivolumab occurred regardless of tumor MCPyV status, with comparable pathologic complete response rates in both virus-positive and virus-negative tumors (28). This suggests that different immunogenic targets are effectively recognized in each tumor type: viral antigens in MCPyV-positive tumors and UV-induced neoantigens in MCPyV-negative tumors. Giraldo et al. demonstrated that both virus-positive and virus-negative MCC tumors exhibit upregulation of the PD-1/PD-L1 axis, potentially explaining the broad efficacy of checkpoint inhibitors across MCC subtypes (45). The convergence of evidence from our study and others demonstrating the significantly poorer prognosis of head and neck MCC relative to other sites of origin, coupled with the promising results of neoadjuvant immunotherapy, provides strong justification for selecting this patient group as a high-priority group for neoadjuvant treatment over surgery first. This approach offers several potential advantages, such as an opportunity for significant tumor downsizing, potentially facilitating less extensive surgery in anatomically sensitive head and neck regions (46). It may also allow for early assessment of treatment response, which has been shown to correlate strongly with long-term outcomes. It may generate more effective systemic immunity against potential micrometastases, addressing the high risk of distant disease in this population (44). These advantages may improve long-term survival in a population with demonstrated poor outcomes under standard treatment approaches. While our study provides valuable long-term outcome data, several limitations warrant acknowledgment. As a retrospective analysis, it is subject to selection bias and potential confounding.

Beside of our suggestions, recently, new therapeutic strategies for treating MCC have been under development. These include targeted therapies such as ponatinib and nilotinib. In addition, preclinical models have demonstrated that the agent m276-SL-PBD can eradicate tumors and induce long-lasting, tumor-free survival. Retinoid-based therapies have also shown promise, further highlighting their potential role in the treatment of MCC (47–49). MCPyV testing was not available for all patients, preventing a direct correlation between biological subtype and outcomes in our cohort. Our cohort spans four decades during which treatment paradigms evolved, potentially affecting outcome interpretation. The sample size, while substantial for this rare tumor, may limit statistical power for subgroup analyses. Additionally, information on surgical margin status and details regarding immunotherapy use after disease recurrence were not uniformly documented. Future prospective studies should evaluate neoadjuvant immunotherapy specifically for head and neck MCC, with stratification by viral status, to definitively establish whether this approach can overcome the inherently poor prognosis of this challenging disease subtype. Development of predictive biomarkers for immunotherapy response, beyond viral status, will be crucial for patient selection. Additionally, investigation of combination therapies that might overcome the aggressive biology of virus-negative tumors is warranted.

## 5 Conclusions

Our findings demonstrate that head and neck location is an independent adverse prognostic factor in local and locally advanced Merkel cell carcinoma, with significantly worse overall survival compared to tumors at other anatomical sites. The strong association between tumor location and MCPyV status, with head and neck tumors being significantly more likely to be virus-negative, provides a possible biological explanation for these outcome differences. Given the consistently poor outcomes observed with head and neck MCC and the promising results from recent clinical trials of neoadjuvant immunotherapy, We suggest that the H&N location should be considered as one of the criteria when selecting patients diagnosed with MCC for neoadjuvant immunotherapy. This recommendation is further supported by the biological characteristics of head and neck MCC, which may render these tumors particularly responsive to immunotherapy despite their overall poor prognosis. Future prospective clinical trials should specifically investigate the efficacy of neoadjuvant immunotherapy in head and neck MCC, ideally with stratification by viral status, to definitively establish whether this approach can overcome the adverse prognosis associated with this location. Until such trials are completed, clinicians should consider the compelling rationale for incorporating neoadjuvant immunotherapy into the management strategy for patients with head and neck MCC, potentially representing a new treatment approach in the treatment of this high-risk disease subtype.

## Data Availability

The raw data supporting the conclusions of this article will be made available by the authors, without undue reservation.
